# SOCIAL EPIGENETICS OF RACIAL DISPARITIES IN AGING

**DOI:** 10.1093/geroni/igac059.1867

**Published:** 2022-12-20

**Authors:** Isabel Yannatos, Sharon Xie, Rebecca Brown, Corey McMillan

**Affiliations:** University of Pennsylvania, Philadelphia, Pennsylvania, United States; University of Pennsylvania, Philadelphia, Pennsylvania, United States; University of Pennsylvania School of Medicine, Philadelphia, Pennsylvania, United States; University of Pennsylvania, Philadelphia, Pennsylvania, United States

## Abstract

Racial disparities in many aging-related health outcomes are persistent and pervasive among older Americans. There are well-documented inequities in social and physical environmental exposures which may contribute to these disparities, but we lack understanding of the biological intermediates by which environmental exposures affect disparate health outcomes. DNA methylation (DNAm) aging captures the residual between biological age, robustly measured by GrimAge and Dunedin Pace of Aging methylation (DPoAm), and chronological age. We hypothesize that neighborhood social environment and air pollution exposures contribute to racial disparities in DNAm aging. We performed retrospective cross-sectional analyses among non-Hispanic participants (N=2611 White, N=639 Black) in the Health and Retirement Study whose 2016 DNAm age is linked to survey responses and geographic data. We observed Black individuals have significantly accelerated DNAm aging on average compared to White individuals according to GrimAge (599%) and DPoAm (498%). We implemented linear regression models and Kittagawa-Blinder-Oaxaca decomposition to identify exposures that contribute to this disparity. Exposure measures include census-tract-level Social Deprivation Index, perceived social stress, particulate matter (PM2.5), nitrogen dioxide, and ozone. Individual-level determinants include socioeconomic status, healthcare access, health status, and health behaviors. Results suggest these individual-level factors account for ~43% of the disparity in GrimAge and ~34% in DPoAm. Higher neighborhood socioeconomic deprivation for Black participants significantly contributes to the disparity in GrimAge, while greater vulnerability to PM2.5 contributes to the disparity in DPoAm. DNAm aging may play a role in the environment “getting under the skin” and contributing to age-related health disparities between Black and White Americans.

